# Low-Dose Collagenase Chemonucleolysis Combined with Radiofrequency in the Treatment of Lumbar Disc Herniation: A 10-Year Retrospective Study

**DOI:** 10.1155/2021/8234558

**Published:** 2021-12-23

**Authors:** Meng Wang, Xuexue Zhang, Yaoping Yu, Gang Xu, Jinping Nie, Bo Yu, Xuezhong Cao, Mizhen Qiu, Yunhua Liao, Daying Zhang, Yi Yan

**Affiliations:** ^1^Department of Pain Medicine, The First Affiliated Hospital of Nanchang University, Nanchang 330006, China; ^2^Graduate School of Jiangxi Medical College, Nanchang University, Nanchang 330006, China; ^3^Department of Pain Medicine, The Ningbo Rehabilitation Hospital, Ningbo 315000, China

## Abstract

**Objective:**

This study explored the 10-year efficacy, safety, and prognostic factors of low-dose collagenase chemonucleolysis (CCNL) combined with radiofrequency (RF) in the treatment of lumbar disc herniation (LDH).

**Methods:**

The data of 167 LDH patients were collected. Modified MacNab criteria, Numerical Rating Scale (NRS), and Japanese Orthopedic Association (JOA) scores were, respectively, used to evaluate patients' excellent and good rates, pain degree, and nerve function. The preoperative and 10-year postoperative patients' pain, numbness, and muscle weakness were compared. Patients' complications in perioperative period, recurrent/reappeared LDH, and reoperations were recorded. Finally, the independent risk factors affecting the long-time efficacy were assessed.

**Results:**

A total of 126 patients were included. The patients' excellent and good rates were 86.51%–92.86% with no significant difference (*P* > 0.05). Postoperative NRS and JOA scores significantly improved (*P* < 0.01), most obvious within 6 months postoperatively. At 10 years postoperatively, 65.08%, 83.95%, and 93.02% of patients' pain, numbness, and muscle weakness were completely relieved (*P* < 0.05). Perioperative complications occurred in three patients with the rate of 2.38%. Recurrent/reappeared LDH patients were 11 with the ratio of 8.73%; nine of them underwent reoperations with the rate of 7.14%. And patients' probability of fair and poor efficacy at 10 years postoperatively with the course of disease >12 months and the responsibility disc ≥2 were, respectively, 6.005 and 4.227 times that of patients with the course of disease ≤12 months and the responsibility disc = 1 (*P* < 0.05).

**Conclusion:**

The combined treatment is effective and safe in the long term. A course of disease *>*12 months and responsibility disc ≥2 independently reduce efficacy, and a course of disease *>*12 months has a more significant impact.

## 1. Introduction

Nearly 80% of the population experiences an episode of low back pain (LBP) at least once during their lifetime [1]. Previous studies have shown that approximately 60% of patients with low back and leg pain were clinically diagnosed with sciatica [2]. Lumbar disc herniation (LDH) is the most common cause of LBP and sciatica, and the mechanism of it mainly involves mechanical compression, inflammatory stimulation, immunological reaction, and pain sensitization [3–5].

Different from open surgeries, minimally invasive surgeries have been gradually applied to clinical LDH treatments because of the advantages of less trauma and strong repeatability [6–8]. Among them, collagenase chemonucleolysis (CCNL) can specifically hydrolyze the main component “collagen type II fibers” in the nucleus pulposus (NP) by collagenase. It degrades collagen type II fibers into amino acids and destroys the NP tissue framework, so that NP tissue is dissolved, absorbed, and shrunk, causing the volume of prominent NP to shrink or even disappear, thus relieving the compression of herniated NP on the nerve roots and dural sac [9, 10]. Since 1970s, CCNL has been used in the clinical treatment of herniated, prolapsed, and dislocated LDH for more than 40 years [11–14]. Currently, radiofrequency (RF) mainly reduces the pressure in the intervertebral disc or herniation by directly denaturing the protein, deconstructing the molecule in NP, and shrinking the NP tissue due to the thermal effect caused by molecular resonance caused by RF electric field. Furthermore, heat energy can also alleviate the inflammatory reaction around the nerve root and the lesion area in the intervertebral disc, destroy the sensory nerve fibers growing into the annulus fibrosus, and block the fissure of the annulus fibrosus. However, because of its limited working range, it has poor effect on giant and prolapsed LDH. So, it is mainly used to treat contained LDH and discogenic LBP in clinic [4, 15–18].

Although the two methods have achieved good clinical effects in the treatment of LDH and discogenic LBP, the associated risks [19], such as aggravated pain, nerve root injury and adverse publicity, disc space infection, anaphylaxis, and cauda equina syndrome (CES), have limited their application and development [20–24], while other studies thought these methods were relatively safe [10, 11, 25]. A study based on the efficacy and safety of CCNL and chymopapain in the treatment of LDH suggested that CCNL may need further study and cannot be recommended at that time [26]. However, the studies on the efficacy and safety of these two are not consistent. For example, compared with CCNL, chymotrypsin chemonucleolysis, a classic chemonucleolysis, was reported to exhibit more adverse reactions such as fatal allergy, paraplegia, death, and causes more injuries to spinal nerve roots and perineural tissue [10, 26–28]. And some studies also found the efficacy of these two was similar [10, 28, 29]. Although chymopapain chemonucleolysis was denied by the FDA because of its insignificant difference compared with placebo in 1976 [30], recently researchers have argued that chymopapain chemonucleolysis (CNL) might be revisited as a treatment option for patients with symptomatic herniated intervertebral discs [21, 26, 29, 31, 32], and that it can still be used safely and effectively as long as patients are carefully selected and an appropriate injection technique is used [33]. More importantly, these studies only suggest that CCNL should be further studied [26, 29]. In 2018, a study confirmed that the optimized digestion of extremely low concentrations of type I and II collagenase combined could save enzymes, was less harmful to NP cells, and was more adapted to separated and cultured NP cells [34]. What is consistent is that, recently, there are still some reports suggesting that CCNL combined with other techniques can be safely and effectively used in the treatment of LDH in journals of North American and European countries [12, 13, 35]. Those studies indicate that CCNL in the treatment of LDH also can be re-emphasized and continued to be researched, improved and applied.

Therefore, it has always been the direction of clinical exploration to reduce the dose of collagenase and the incidence of perioperative adverse events as much as possible, improve safety, fully hydrolyze herniations, and make up for the shortcomings of small working range of RF.

Previous researchers [14] have confirmed that RF can avoid the increased pressure resulting from CCNL by reducing collagenase activity and excessive hydrolysis of collagenase-induced herniated NP on porcine. At the same time, they confirmed that low-dose CCNL combined with RF displayed good short-term effects (3 months) in LDH patients [14]. Moreover, another study also confirmed that low-dose CCNL combined with RF was effective and safe for the treatment of cervical intervertebral disc herniation (1 year) [36]. However, there was no report on the long-term effects on cervical and LDH.

In this study, the 10-year efficacy, safety, and prognostic factors of low-dose CCNL combined with RF in the treatment of LDH were conducted by a retrospective approach. These data may provide a reference point for the clinical application of CCNL combined with RF in the treatment of LDH.

## 2. Materials and Methods

### 2.1. Data Collection and Follow-Up

**Figure 1:**
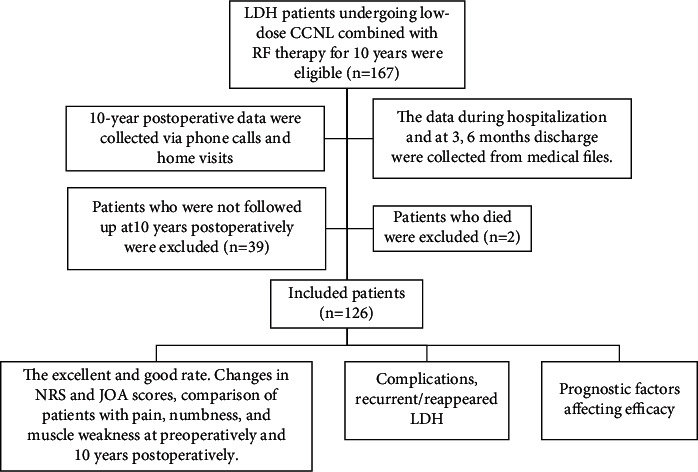
Study design and procedure.

This retrospective study was approved by the Ethics Committee of the First Affiliated Hospital of Nanchang University, and all patients were informed about the study and their consent was obtained. Data of those LDH patients who underwent low-dose CCNL combined with RF in the Pain Department of the First Affiliated Hospital of Nanchang University between June 2009 and June 2010 during hospitalization and at 3 and 6 months after discharge were collected from medical files. In addition, 10-year postoperative data were collected via phone calls and home visits upon follow-up. Patients who missed three calls or one home visit were considered lost to follow-up (Figure 1).

### 2.2. Inclusion and Exclusion Criteria

All patients who underwent an operation for symptomatic LDH by low-dose CCNL combined with RF were eligible for the study.

Inclusion criteria were as follows: (1) obvious radicular pain; (2) magnetic resonance imaging (MRI) confirmation of segmental disc herniation corresponding to the leg pain or secondary nonsevere spinal canal and lateral recess stenosis and computed tomography (CT) confirmed no calcification of the herniation; and (3) positive lumbar discography and pain provocation test.

Exclusion criteria were as follows: (1) allergic to collagenase; (2) only LBP; (3) severe cardiovascular and cerebrovascular diseases; (4) same-segment protrusion and lumbar spondylolisthesis; (5) age > 75 years; and (6) died during follow-up.

### 2.3. Surgical Technique

**Figure 2:**
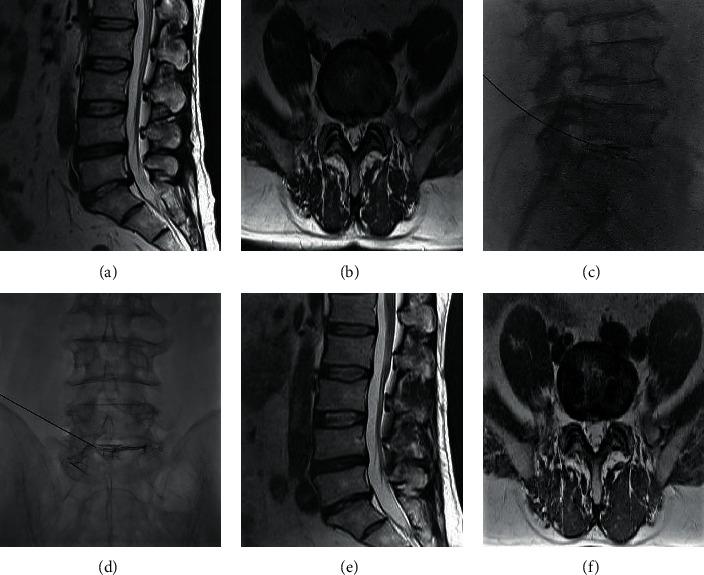
Low-dose CCNL combined with RF for LDH. (a, b) Patients lumbar MRI at preoperatively: L5S1 disc herniated. (c, d) Intraoperative X-ray radiography: contrast agent diffuses during operation. (e, f) Patients lumbar MRI at 10 years postoperatively: the herniation of L5S1 disappeared.

It was first ensured that the RF electrode's working end could be completely inserted into the protrusions after the puncture needle reached them, and then appropriate RF electrodes with different working ends were chosen. The target intervertebral spaces were located and marked, the puncture angle and length were measured under CT guidance, and the operation area was sterilized and disinfected. When the puncture reached the target anatomical area, 0.3–0.5 mL of contrast agent (iohexol; GE (Shanghai) Pharmaceutical Industry, Shanghai, China) was injected, and the diffusion pattern was observed. The protrusion imaging was duplicated as clearly as possible. If the contrast agent diffused more than 7 mm away from the electrode zero point, 50−200 U/0.1–0.4 mL of collagenase (Liaoning Wei Bang, Biological Pharmaceutical Co, LTD, Liaoning, China) was injected. After the RF electrode was positioned, it was connected to the RF instrument (Beijing Bei Qi Technology Medical Company, Beijing, China). After the electrical impedance was measured (the electrical impedance of the NP was generally 200–400 Ω), the sensory-evoked (100 Hz, 0.5–1.0 mA) and motion-evoked impedance (3 Hz, 1.0–2.0 mA) were measured. Failure to induce pain in the lower limb muscle during contraction was considered as evidence that the electrode was distant from the nerve root. The initial working parameter was set at 70°C/60 s to conduct single consecutive radiofrequency thermocoagulation, after which the parameter was increased stepwise to 80°C/60 s, 85°C/60 s, and 90°C/60 s. The LBP and the sensation of warmth could then be duplicated, but electric shock-like numbness or pain was avoided if possible. It was reconfirmed that the localization of the electrode was on the target, far away from the nerve root, after which the parameters were set at 95°C/90 s and continued for two cycles. If there were ≥2 responsibility discs, the operation was continued according to the above steps. Throughout the procedure, communication with the patient was maintained, with close attention paid to the status of the patient's pain and nerve function of the lower extremities (Figure 2).

### 2.4. Postoperative Management

Patients were conveyed to the hospital ward postoperatively and administered antibiotics for 3 days. After lying supine on a bed conventionally for 7–10 days, the patients could get up with a waistband. Appropriate back muscle exercises were performed, and manual labor was prohibited for 3 months.

### 2.5. Evaluation Indices

The modified MacNab criteria [37] were used to evaluate the excellent and good rate of patients at 3, 6, and 120 months postoperatively. Because the postoperative relative bed rest was 3 months, the evaluation was not started until 3 months postoperatively. NRS and JOA scores were used to evaluate the degree of pain and nerve function, respectively (preoperatively, immediately postoperatively, discharge, and 3, 6, and 120 months postoperatively). Because patients needed to lie supine on a bed for 7−10 days postoperatively, there was no immediate postoperative JOA evaluation. Preoperative and 10-year postoperative patients with pain, numbness, and muscle weakness were compared, and complications in perioperative period, recurrent/reappeared LDH, and reoperations were recorded. Finally, independent risk factors affecting prognosis were assessed.

### 2.6. Statistical Analysis

All data were analyzed using SPSS version 20.0 (SPSS Inc., Chicago, IL, USA). Categorical variables are expressed in numbers and proportions, and a chi-square test or Fisher's exact test was used for respective comparisons. Continuous variables are expressed as the mean ± standard error of the mean $\left(\bar{x}\pm \text{SEM}\right)$ or interquartile range. In comparing the NRS and JOA scores at different times pre- and postoperatively, if the data conformed to normality and homogeneity of variance, one-way ANOVA was used in combination with Tukey's multiple comparison test, and if data did not conform to normality and homogeneity, the Kruskal–Wallis test was used in combination with Dunn's multiple comparison test. In analyzing prognostic factors, univariate analysis was performed first to compare the differences of factors (gender, age, BMI, etc.) between patients with fair and poor effect and patients with excellent and good effect by the chi-square test. Then, the factor with *P* < 0.1 in the results of the univariate analysis was used as an independent variable, and the modified MacNab was used as a dependent variable for the binary logistic multiple factor regression analysis, and the OR and its 95% confidence interval (CI) were calculated. *P* < 0.05 was considered statistically significant.

## 3. Results

A total of 167 patients were enrolled in this study, and 128 patients were followed up at 10 years postoperatively, during which 2 patients died and were excluded. Therefore, 126 patients were included.

### 3.1. General Information

A total of 84 men and 42 women were included in this study. The average age was 44.27 years (15–73). The average BMI was 24.36 kg/m^2^ (17.62–28.68). The average course of the disease was 35.72 months (1–250). Specific results are shown in Table 1.

### 3.2. Clinical Efficacy

#### 3.2.1. The Excellent and Good Ratio Assessed by Modified MacNab Criteria at Different Times Postoperatively

**Figure 3:**
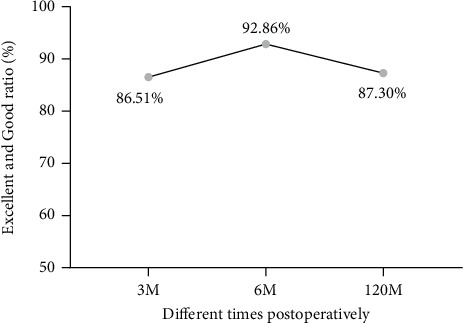
The excellent and good rate of patients was assessed by the modified MacNab criteria beginning at 3 months postoperatively. There were no significant differences between them (*P* > 0.05).

The excellent and good rates were 86.51%, 92.86%, and 87.30% at 3 months, 6 months, and 120 months postoperatively, respectively, and there were no significant differences between them (*P* > 0.05, Figure 3).

#### 3.2.2. Changes in Pre- and Postoperative NRS and JOA Scores

**Figure 4:**
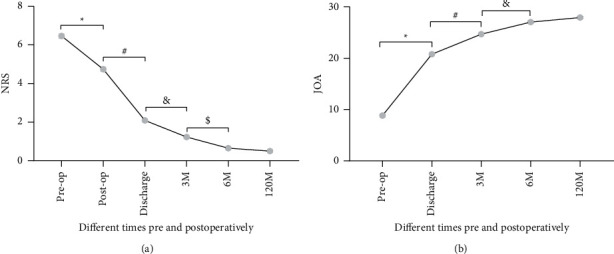
Changes in the pre- and postoperative NRS (a) and JOA (b) scores. NRS (a) showed a decreasing trend, and JOA (b) showed an increasing trend. The postoperative NRS and JOA scores were significantly improved compared with preoperative values (*P* < 0.01), but the improvements were most obvious within 6 months postoperatively. During 6 months postoperatively, NRS and JOA scores at each follow-up time improved significantly compared with the previous follow-up value (*∗*, #, &, $: *P* < 0.01). However, after 6 months postoperatively, NRS and JOA scores did not change significantly (*P* > 0.05).

Postoperative NRS and JOA scores significantly improved compared with preoperative scores (*P* < 0.01), but the improvements were most obvious within 6 months postoperatively. During 6 months postoperatively, the NRS and JOA scores at each follow-up time improved significantly compared with those at the previous follow-up (*P* < 0.01). The NRS score improved from a preoperative value of 6.46 ± 0.12 to a 6-month postoperative score of 0.65 ± 0.06, and the JOA score improved from 8.81 ± 0.25 to 27.02 ± 0.19 points. However, after 6 months, there were no further significant differences in the changes in NRS and JOA scores (*P* > 0.05, Figures 4(a), 4(b)).

#### 3.2.3. Preoperative and 10-Year Postoperative Comparison of Patients with Pain, Numbness, and Muscle Weakness

**Figure 5:**
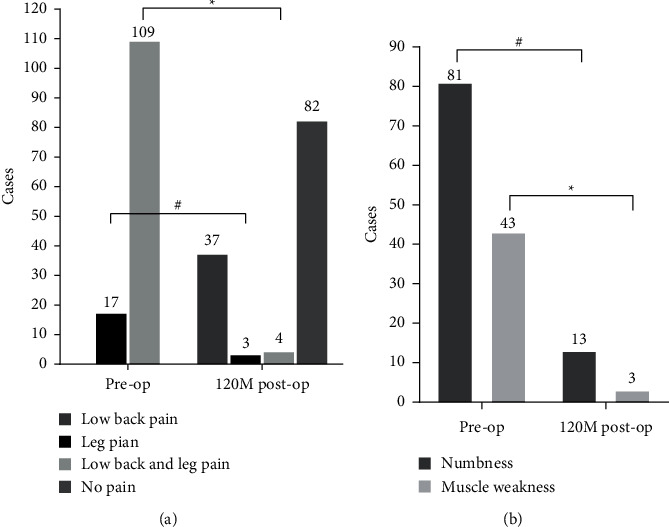
Comparison of patient cases with pain (a) and numbness and muscle weakness (b) at preoperatively and 10 years postoperatively. There were significantly fewer cases with low back and leg pain and leg pain alone (a) and numbness and muscle weakness (b) at 10 years postoperatively compared with the number of preoperative cases (*∗*, #: compared with preoperative values, *P* < 0.05).

Preoperatively, 86.51% and 13.49% of patients experienced low back pain (109/126) and leg pain (17/126), respectively. At 10 years postoperatively, 65.08% of patients (82/126) experienced no pain, only 3.17% of patients (4/126) experienced both low back and leg pain, 2.38% of patients (3/126) experienced leg pain alone, and 29.37% of patients (37/126) experienced residual low back pain. 64.29% of patients (81/126) suffered lower extremities numbness preoperatively, which reduced to 10.32% of patients (13/126) at 10 years postoperatively; accordingly, 83.95% of patients (68/81) experienced complete relief of numbness (*P* < 0.05, Figures 5(a), 5(b)). What's more, 34.13% of patients (43/126) had lower extremities muscle weakness preoperatively, while only 2.38% of patients (3/126) still had muscle weakness at 10 years postoperatively. Overall, 65.08% (82/126), 83.95% (68/81), and 93.02% (40/43) of LDH patients' pain, numbness, and muscle weakness were completely relieved at 10 years postoperatively (*P* < 0.05, Figures 5(a) and 5(b)).

### 3.3. Perioperative Complications and the Treatments of Recurrent/Reappeared LDH

Perioperative complications occurred in 3 patients, and the incidence of complications was 2.38%. One of the three patients, with CES, underwent emergency surgery and completely relieved the symptoms. The other 2 patients developed aggravated LBP within 1 week postoperatively, and the symptoms gradually disappeared after analgesic treatment, dehydration, and nerve nutrition. None of the three patients had sequelae at the 10-year follow-up.

**Figure 6:**
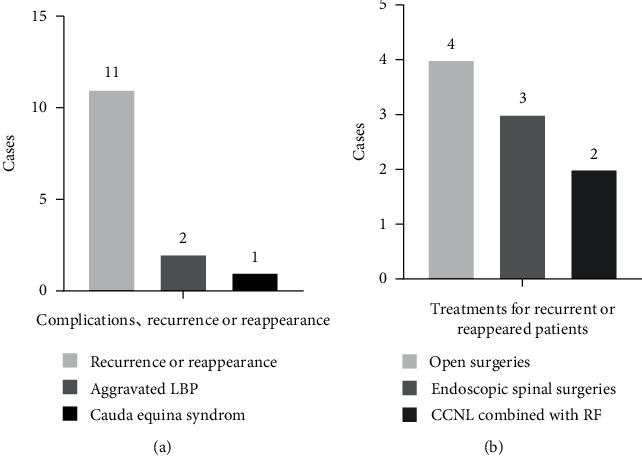
(a) Complications, recurrence, or reappearance. (b) Treatments for recurrent or reappeared LDH patients.

Moreover, 11 patients suffered recurrent/reappeared LDH with a rate of 8.73%. Among them, 2 patients underwent conservative treatments and 9 patients underwent reoperations with a reoperation rate of 7.14%. Among the 9 patients who underwent reoperations, 2 patients underwent CCNL combined with RF, 4 patients underwent open surgeries, and 3 patients underwent spinal endoscopic surgeries (Figures 6(a), 6(b)).

### 3.4. Univariate Analysis and Binary Logistic Multivariate Regression Analysis

At 10 years postoperatively, 110 patients exhibited excellent and good efficacy, and 16 exhibited fair and poor efficacy. Among the 16 patients with fair and poor efficacy, 68.75% of patients (11/16) had a BMI *>*25 kg/m^2^, 81.25% of patients (13/16) had a course of disease *>*12 months, and 50% of patients (8/16) had responsibility disc ≥2. In contrast, among the 110 patients with excellent and good efficacy, 42.73% of patients (47/110) had a BMI *>*25 kg/m^2^, 37.27% of patients (41/110) had a course of disease *>*12 months, and 14.54% of patients (16/110) had responsibility disc ≥2 (Table 2).

#### 3.4.1. Univariate Analysis

Univariate analysis showed that the proportion of BMI >25 kg/m^2^, course of disease >12 months, and responsibility disc ≥2 in patients with fair and poor efficacy was significantly higher than that in patients with excellent and good efficacy (Χ^2^ = 3.847, *P* = 0.050; Χ^2^ = 11.364, *P* = 0.001; Χ^2^ = 9.278, *P* = 0.002, respectively, Table 2).

#### 3.4.2. Binary Logistic Multivariate Regression Analysis

**Figure 7:**
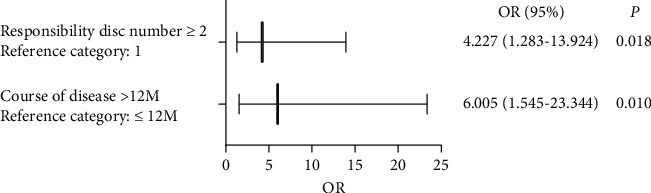
Results of the binary logistic multivariate regression analysis. *P* < 0.05 indicates that the factor can reduce the efficacy alone. BMI was eliminated using the stepwise regression equation.

Binary logistic multivariate regression analysis showed that the probability of fair and poor efficacy with the course of disease >12 months was 6.005 times greater than that with the course of disease ≤12 months (OR = 6.005, 95% CI: 1.545–23.344, *P*=0.01, Figure 7), and the probability of fair and poor efficacy with the responsibility disc ≥2 was 4.227 times greater than that with responsibility disc = 1 (OR = 4.227, 95% CI: 1.283–13.924, *P*=0.018, Figure 7). According to this analysis, BMI was eliminated by stepwise regression, suggesting that BMI does not affect the treatment efficacy. Therefore, a course of disease *>*12 months and responsibility disc ≥2 were determined to be independent risk factors reducing treatment efficacy (Figure 7).

## 4. Discussion

When CCNL and RF are used to treat LDH, how to reduce the dose of collagenase, make up for the shortcomings of the small working range of RF electrode, and improve the clinical efficacy, so that these classic minimally invasive techniques can continue to be safely and effectively applied to LDH treatment, has always been the direction of clinical exploration. Zhang et al. [14] used low-dose CCNL combined with RF in porcine intervertebral discs, which showed that RF reduced the amount of NP hydrolyzed induced by CCNL, as well as the content of hydroxyproline and glycosaminoglycan. Combining with their clinical study (3 months), they reported that targeted RF combined with CCNL was an effective and safe method for the treatment of LDH. Furthermore, another study has also confirmed that the short-term efficacy of low-dose CCNL combined with RF in cervical disc herniation was effective and safe [36].

In our study, the excellent and good rate was 86.51% at 3 months postoperatively. While it was 88.10% in Zhang's [14] study, and there was no significant difference between them. And it was 92.86% at 6 months postoperatively. Compared with the study of Zhang, the lowest dose of collagenase (50 U) in our study was lower than that in theirs (70 U), and the RF parameters were similar. Thus, our study can be considered a supplement and extension of their research. Most importantly, the excellent and good rate at 10 years postoperatively in our study was 87.30%, which was high as a long-term efficacy compared with the 5-year efficacy of CCNL on LDH of 52% [26]. We believe that not only related to adhering strictly to clinical indications avoiding surgical injury, but more importantly, according to Zhang's study [14], low-dose collagenase injection may avoid excessive degradation of collagen in the NP, lower the possibility of surgical-related intervertebral disc degeneration, and permit RF to repair the fibrous annulus to protect the structural integrity and stability of the intervertebral disc. So, our study indicated that the improvement in postoperative patients was stable and good.

NRS and JOA scores in our study improved most significantly were within 6 months postoperatively, and patients reached a state of basically painless and recovered nerve function at 6 months postoperatively. It may be related to lying supine on a bed and forbidding physical activities for 3 months postoperatively, causing poor back muscle strength, lumbar mobility, and stability, resulting in discomfort after bending over and sedentary activities, and therefore, patients still felt pain and neurological dysfunction after appropriate bending and other activities. While from 3 months postoperatively, patients gradually returned to normal life and work, and the strength of lumbar back muscles, lumbar mobility, and stability gradually recovered, by 6 months postoperatively, patients basically were painless and nerve functions basically were normal. It suggested that when using low-dose CCNL combined with RF to treat LDH, instructing patients to take exercise the back muscles properly to avoid overprotection is necessary.

We found most patients suffered from low back and leg pain preoperatively, while 65.08% (82/126) patients were completely painless at 10 years postoperatively, which was consistent with the conclusion that CCNL had a good effect on sciatica caused by LDH in previous studies [24, 28]. However, at present, there are few studies about the numbness recovery of LDH patients treated by surgeries, and most of them think that postoperative numbness recovery is slow or even persistent [38–40], which is mainly related to the damage of large-diameter myelinated nerve fibers, deformation of nerve fibers, demyelination, and slow regeneration of nerve axons [38]. In our study, 83.95% (68/81) of patients were completely relieved at 10 years postoperatively, which was significantly higher than that reported in previous studies [38, 41], and we think this was mainly related to the long follow-up time. In addition, there are also few studies on muscle strength recovery after LDH surgeries and some of them have shown that nearly 75% of LDH patients' muscle weakness can be completely recovered within 1 year postoperatively, while they think that the recovery of postoperative muscle strength is mainly related to the degree of preoperative muscle weakness, but inconsistent with the results of various studies on the duration of preoperative muscle weakness [42–45]. Mariconda et al. [43] think that even if the short-term postoperative feeling and muscle strength recovery are not good, patients can still expect long-term satisfactory feeling and muscle strength recovery as long as the short-term postoperative pain can be obviously relieved. In our study, 34.12% of patients (43/126) had muscle weakness preoperatively, while 93.02% of patients (40/43) had completely recovered muscle strength at 10 years postoperatively, which was significantly higher than previous research results. This may be related to the low degree of preoperative muscle weakness (≥grade 2) and the long-term follow-up time. What is more, although, at 10 years postoperatively, there were still patients with pain, numbness, and muscle weakness, the number, degree, and range were significantly reduced compared with those at preoperatively and most patients with discomfort were satisfied with surgeries. As a result, our study showed that combination can significantly improve the pain, numbness, and muscle weakness of LDH patients, improving their life quality.

Complications such as chemical radiculitis, CES, severe allergies, and death may occur when LDH patients are treated with CCNL [24, 46, 47]. However, the incidence of fatal anaphylaxis, paraplegia, and other serious adverse reactions were significantly lower than that of chymotrypsin chemonucleolysis in some studies [10, 26, 28]. The long-term efficacy of RF on LDH may be poor, and there is a high risk of nerve root injury during RF [17, 48]. What is exciting is that, in recent years, with the continuous optimization of the CCNL injection method, dosage, and purity, no serious complications such as paraplegia and death were reported. In this study, 3 patients had perioperative complications with a 2.38% of complication rate, which was not higher than the previously reported complications rate of CCNL and RF for LDH treatment [16, 49].

One patient developed CES after coughing at 24 hours postoperatively, and the other 2 patients had aggravated LBP within 1 week postoperatively, but the muscle weakness and numbness were not when they got up on their own.

According to previous research, CCNL may increase the pressure in the lumbar disc and aggravate the nerve compression and the most obvious within one week, which is the main risk of CCNL [14, 47]. And the incidence of sciatica and back pain after low-dose CCNL was significantly lower than that with high dose [28]. So, E. Wintermantel et al. suggested that reducing the dose of collagenase to make the relation between enzyme activity and disc volume more appropriate can reach better and more reliable results [47]. Although some studies questioned that CES and aggravated LBP were related to CCNL's digestive damage to surrounding tissues such as annulus fibrosus, end-plates, and bone [22], which also have been questioned and denied by other studies [10, 11, 25, 28], there is no conclusion yet. And some poor efficacy of CCNL in LDH was a diagnosis error [11]. And patients got up and took activities too early postoperation in a previous study [11]. Therefore, in our research, we try to reduce the occurrence of CCNL complications by the following methods. Firstly, we strictly grasped the indication, such as patients with stenosis of the spinal canal or intervertebral foramen caused by larger protrusion had been excluded. Secondly, we used a lower more dose (50–200 U) similar to the study of Zhang et al., and there was no CES occurred in that study [14]. Thirdly, we injected collagenase first and then carried out RF in the process. Use RF to inactivate part of collagenase in the disc and repair the needle channel and fissure and annulus fibrosus, thus reducing the possibility of collagenase dissolving normal nucleus pulposus tissue and nucleus pulposus reherniation. Finally, we were very cautious about postoperative activities; thus we told patients to stay in bed for 7–10 days, then they could get up with a waistband and perform appropriate back muscle exercises, moreover, manual labor was prohibited for 3 months.

Although there was one patient with CES and two patients with aggravated LBP in our study, which occurred within 24 hours after coughing and self-getting up, respectively, we do not think the CES was directly caused by low-dose CCNL, but it was due to the increased abdominal pressure caused by severe cough, leading to a further increase in pressure in the disc and spinal canal where the pressure relatively increased after CCNL, which resulted in herniated NP and nerve compression. In addition, although we have made strict postoperative instructions, some patients would still fail to follow or have uncontrollable reasons as the patient with CES coughed, which would inevitably lead to adverse events. We believe a similar situation may exist in any operations, not only in ours, such as in the chymopapain chemonucleolysis [28, 32], microdiscectomy, and decompressive laminectomy [50, 51]. For example, a study of Wardlaw et al. in 2013 [32], a prospective randomized trial of chemonucleolysis compared with surgery for soft LDH with short- and long-term outcomes, showed that chemonucleolysis (chymopapain) was as effective as surgery and with fewer complications, and they believed that restoration of its availability would be beneficial to patients even though there was a CES in the chymopapain group over the 24 hours following treatment. When it is relatively consistent, in those studies, patients with CES recovered completely. Like them, we were highly sensitive to CES, so we immediately asked the orthopedic consultation for open surgery, and the patient recovered very well after surgery. Moreover, CES may occur and need to be remedied by open surgery, which would allow patients to get informed of consent preoperation. And two patients with aggravated LBP also recovered very well after conservative treatments. Chymopapain chemonucleolysis in the treatment of lumbar disc herniation would also cause CES, which can still be re-studied and used. Therefore, we believe it is true of CCNL. Considering all these, we believe that the low-dose CCNL combined with RF is safe relatively.

In addition, 8.73% of patients (11/126) suffered recurrent/reappeared LDH, and the reoperation rate was 7.14% (9/126). In addition to open and spinal endoscopic surgeries, there were still patients who continued to undergo low-dose CCNL combined with RF, which indicated that the initial low-dose CCNL combined with RF did not affect the way of reoperation and was repeatable, which was consistent with previous studies [21, 31]. In summary, the surgery was very safe and protected the integrity of the intervertebral disc and surrounding tissues.

In our study, in order to improve the efficacy and safety and reduce complications of CCNL, we not only reduced the dose of collagenase and combined it with RF but also adopted the special operation procedure of injecting collagenase before RF. Why did we do so, there were three purposes. First, the dose of collagenase can be further reduced by inactivating part of collagenase by RF. Second, after collagenase injection, it would spread along the fissure in normal NP, which might overdegrade the normal NP, and RF could repair these cracks to some extent reducing the dissolution of collagenase to normal NP. Third, RF can repair the annulus fibrosus to a certain extent, reduce the possibility of reherniated nucleus pulposus, and improve the surgical effect. However, some people may question whether the injected collagenase was inactivated completely by following RF making collagenase did not work. First of all, this question is reasonable. Secondly, our results confirmed that collagenase was indeed partially inactivated, not completely. Explanations are as follows: First, if collagenase was completely inactivated, RF alone would not achieve good and stable efficacy in the short- and long-term as in our study. Because the current indication of RF is lumbar discogenic pain, while some studies think that it is doubtful about its long-term efficacy perhaps because of its limited working scope. On the contrary, in our research, eligible subjects were all herniated LDH, and patients had good and stable short- and long-term efficacy, showing that CCNL played a major role. Secondly, from the point of view of adverse effects, there were no obvious adverse effects such as loss of intervertebral disc height, indicating that CCNL had not overdegraded normal NP. Finally, the results of our study indicate the dosage of collagenase is moderate and this operation process is reasonable and safe.

At present, there is no consensus on the related factors affecting the efficacy of LDH surgeries, such as sex, age, BMI, smoking, and endplate Modic changes on prognosis varied across studies [52, 53]. In this study, all the factors mentioned in previous studies affecting the efficacy were included [54]. In fact, the responsibility disc in this study was not always single, and there may have been multiple combinations on the degeneration grade of the disc, spinal canal morphology, Modic changes, etc. Therefore, these factors were excluded. This study showed that the course of disease >12 months and the responsibility disc ≥2 were independent risk factors reducing efficacy, and the former had a more obvious influence. We believe that a longer course of disease or a greater number of responsibility discs raise the risk of poorer postoperative efficacy, and the course of disease has a more significant effect on the efficacy, which is consistent with previous studies [52–56].

Previous studies have demonstrated that a longer disease duration predicts a worse outcome [54–57]. Hong's study [54] reported that patients with sciatica >12 months had a less favorable outcome, which was consistent with our study results. We believe that the influence of the course of disease on efficacy is mainly related to the following three points: Firstly, a longer disease duration signifies longer compression of the nerve roots, which may result in an irreversible lesion, resulting in greater difficulty in treating the condition [54], and increased risk of numbness [41], which view is widely reported and accepted at present. Secondly, a longer disease duration may cause central sensitization of pain. For instance, in a rat model, a study [58] confirmed that central sensitization was involved in radicular and chronic pain in LDH. Moreover, using functional MRI, Zhou [59] showed that in nonspecific LBP patients with a longer course of the disease, the structure and function of the corresponding brain regions would change, causing central sensitization pain [60]. Furthermore, Dolgun et al. [61] suggested that antiepileptic drugs such as gabapentin and pregabalin were effective for patients with early postsurgical neuropathic pain after LDH surgery. However, we think that the pain described in Habibullah's study is more inclined to the central sensitization pain proposed by Nijs et al. in 2014 [60], although it has not been widely accepted yet. Nijs suggested that the treatment should mainly focus on the central nervous system [60]. Thirdly, a longer course of disease easily causes anxiety and depression, which are independent risk factors for poor postoperative efficacy [54, 56, 62]. For example, Haugen et al. [57] considered that psychosocial factors were more important to the long-term prognosis of LDH patients than the specific symptoms and dysfunction of sciatica preoperatively.

Although this study has not clearly diagnosed whether patients had central sensitization, anxiety, and depression, the course of disease >12 months was an independent risk factor affecting the efficacy, indicating that this may be influenced by central sensitization and psychological factors. In this study, most patients with a course of disease >12 months had long histories of chronic LBP. Some patients with a fair and poor efficacy had LBP >10 years, and they reported significantly more discomfort. These patients were still reluctant to bend over, sitting, and lying down for extended periods, and they reported that when they bent over, they felt obvious discomfort in the back and legs; nevertheless, lumbar MRI showed no signs of nerve root compression or obvious disc degeneration. They repeatedly asked doctors if there were any problems with the lumbar MRI and why they still felt pain, showing obvious anxiety and depression emotions. Based on previous studies, we speculated that these patients may be accompanied by anxiety and depression. Although the nerve root compression and inflammation had been relieved, the efficacy was not apparent. Therefore, in such patients, the conservative treatment time can be shortened [54] and the patients can be screened for central sensitization pain and treated for it [60]. There is a need for health education for LDH surgical patients to encourage them to have a positive attitude, and appropriate exercises will also contribute to a good prognosis [54, 57].

At present, multidisc operations for LDH are rare, and there is no research exploring whether the number of responsibility disc affects the prognosis of surgery. However, studies indicate that severe intervertebral disc degeneration is a risk factor that affects the efficacy of LDH surgery [54], rather than the size of the protrusion [63]. Moreover, it is believed that severe degeneration of the adjacent intervertebral disc is also a risk factor affecting the prognosis [54]. Consequently, we hypothesize that a higher number of responsibility discs would be related to greater severity of degeneration of the lumbar and thus result in poorer postoperative efficacy. Other factors such as BMI, sex, smoking, and postoperative strenuous activities were not found to significantly affect efficacy, which was not contradictory to previous studies.

Recently, the use of CCNL in LDH has been gradually reduced due to all kinds of reasons. However, more studies now suggest that CNL can be an effective method for treating LDH [31–33] and may be able to reduce the number of patients who undergo spinal surgeries and thus the inherent morbidity and mortality involved with all surgical procedures [31]. Moreover, CCNL combined with injection of oxygen-ozone [12] or psoas compartment block [13] also can be regarded as a useful treatment for LDH. And a study [64] suggested that the very-low-concentration collagenase digestion method was used to obtain high-purity and sufficient NP cells, which was less harmful to NP cells and thus may help improve the efficacy and safety of the method. We believe that by strictly adhering to clinical indications, improving the clinical skills of doctors, and rigorously abiding by postoperative rehabilitation advice, low-dose CCNL combined with RF remains an effective solution for LBP caused by LDH. This may be useful in grassroots and community hospitals where there is a lack of resources to properly conduct spinal endoscopy. It may also be a better option for the majority of patients who do not want to undergo spinal endoscopy and open surgery [65].

Our study has limitations. First of all, this study is a single-center retrospective study, lacking control group. There are some unrealizable factors. According to previous studies, CCNL has not been used alone in the treatment of LDH, and RF is mainly used for the treatment of discogenic LBP, nor can it be used alone in the patients included in this study. Moreover, since 2009, more medical records could be found in our medical files, and the previous records had not been imported into the system. Secondly, the follow-up time span of this study is long, and the continuity of follow-up data is poor. Therefore, in future studies, multicenter prospective controlled studies can be carried out, which will provide a more comprehensive basis for the efficacy, safety, and prognostic factors of low-dose CCNL combined with RF in the treatment of LDH.

## 5. Conclusions

We believe that low-dose CCNL combined with RF is a safe and effective method for treating LDH, with good and stable long-term efficacy and no serious complications. What is more, a course of disease >12 months and responsibility disc ≥2 are independent risk factors reducing efficacy, and a course of disease >12 months has a more obvious effect. We hope our study can provide a reference for the clinical treatment of LDH. And in clinical treatment of LDH patients with low-dose CCNL combined with RF, it is necessary to fully consider the risk factors that affect the efficacy and formulate an individualized diagnosis and treatment plans for patients.

## Figures and Tables

**Table 1:** Patients' characteristics.

Patients' characteristics	Classification	Cases $\left(\bar{x}\pm \text{SEM}\right)$	Percentage (%)
Sex	M/F	84/42 44.27 ± 1.03	66.67/33.33
Age(Y)	≤50/>50	87/39 24.36 ± 0.18	69.05/30.95
BMI (kg/m^2^)	≤25/>25	68/58 35.72 ± 4.85	53.97/46.03
Course of disease (M)	≤12/>12	72/54	57.14/42.86
Preoperative pain location	Low back and leg pain/leg pain	109/17	86.50/13.50
Preoperative numbness	No/yes	45/81	35.71/64.29
Preoperative muscle weakness	No/yes	83/43	65.87/34.13
Number of responsibility disc	1/≥2	102/24	80.95/19.05
Preoperative NRS	4–6/7–10	6.46 ± 0.12 64/62	50.79/49.21
Preoperative JOA	≤10/11–15	8.81 ± 0.25 70/56	55.56/44.44
Smoking history	No/yes	68/58	53.97/46.03
Postoperative prolonged standing, sedentary, and bending over	No/yes	45/81	35.71/64.29
Postoperative engaging in physical works	No/yes	86/40	68.25/31.75
Postoperative strenuous activities	No/yes	110/16	87.30/12.70
Lumbar trauma history	No/yes	121/5	96.03/3.97
Lumbar surgery history	No/yes	117/9	92.86/7.14
Diabetes history	No/yes	120/6	95.24/4.76

Note: All factors were transformed into binary variables.

**Table 2:** Results of univariate analysis.

Patient' characteristics	Classification	Cases	Excellent and good	Fair and poor	*χ * ^2^	*P* value
Sex	Male	84	71	13	1.912	0.167
	Female	42	39	3		
Age (Y)	≤50	87	76	11	0.001	0.978
	>50	39	34	5		
BMI (kg/m^2^)	≤25	68	63	5	3.847	0.050^**#**^
	>25	58	47	11		
Course of disease (M)	≤12	72	69	3	11.364	0.001^**#**^
	>12	54	41	13		
Preoperative pain location	Low back and leg pain	109	94	15	0.975	0.323
	Leg pain	17	16	1		
Preoperative numbness	No	45	37	8	1.571	0.092
	Yes	81	73	8		
Preoperative muscle weakness	No	83	73	10	0.092	0.762
	Yes	43	37	6		
Number of responsibility disc	1	102	94	8	9.278	0.002^**#**^
	≥2	24	16	8		
Preoperative NRS	4–6	64	58	6	1.306	0.253
	7–10	62	52	10		
Preoperative JOA	≤10	70	59	11	0.173	0.677
	11–15	56	51	5		
Smoking history	No	68	63	5	0.544	0.461
	Yes	58	51	7		
Postoperative prolonged standing, sedentary, and bending over	No	81	68	13	2.512	0.113
	Yes	45	52	3		
Postoperative engaging in physical works	No	86	73	13	1.552	0.213
	Yes	40	37	3		
Postoperative strenuous activities	No	110	12	98	2.106	0.147
	Yes	16	12	4		
Lumbar trauma history	No	121	106	15	0.220	0.639
	Yes	5	4	1		
Lumbar surgery history	No	117	103	14	0.679	0.410
	Yes	9	7	2		
Diabetes history	No	120	105	15	0.083	0.773
	Yes	6	5	1		

Note: Chi-square values and *P* values of the univariate analysis were calculated to compare the relationship between each factor and the therapeutic effect. #: *P* < 0.1, the corresponding factors were subjected to binary logistic multivariate regression analysis.

## Data Availability

The data used to support the findings of this study are available from the corresponding author upon request.

## Abbreviations

CCNL: Collagenase chemonucleolysis
CNL: Chemonucleolysis
RF: Radiofrequency
LBP: Low back pain
LDH: Lumbar disc herniation
NRS: Numerical Rating Scale
JOA: Japanese Orthopedic Association
CES: Cauda equina syndrome
NP: Nucleus pulposus
$\bar{x}$: Mean
SEM: Standard error of mean
BMI: Body mass index
OR: Odds ratio
M/F: Male/Female.
